# The maternal uniparental disomy of chromosome 6 (upd(6)mat) “phenotype”: result of placental trisomy 6 mosaicism?

**DOI:** 10.1002/mgg3.324

**Published:** 2017-09-22

**Authors:** Thomas Eggermann, Barbara Oehl‐Jaschkowitz, Severin Dicks, Wolfgang Thomas, Deniz Kanber, Beate Albrecht, Matthias Begemann, Ingo Kurth, Jasmin Beygo, Karin Buiting

**Affiliations:** ^1^ Medical Faculty Institute of Human Genetics RWTH Aachen University Aachen Germany; ^2^ Praxis für Humangenetik Homburg Germany; ^3^ Klinikum Mutterhaus der Borromäerinnen Trier Germany; ^4^ Institute of Human Genetics University of Essen Essen Germany

**Keywords:** Chromosome 6, imprinting disorder, trisomic rescue, uniparental disomy

## Abstract

**Background:**

Maternal uniparental disomy of chromosome 6 (upd(6)mat) is a rare finding and its clinical relevance is currently unclear. Based on clinical data from two new cases and patients from the literature, the pathogenetic significance of upd(6)mat is delineated.

**Methods:**

Own cases were molecularly characterized for isodisomic uniparental regions on chromosome 6. For further cases with upd(6)mat, a literature search was conducted and genetic and clinical data were ascertained.

**Results:**

Comparison of isodisomic regions between the new upd(6)mat cases and those from four reports did not reveal any common isodisomic region. Among the patients with available cytogenetic data, five had a normal karyotype in lymphocytes, whereas a trisomy 6 (mosaicism) was detected prenatally in four cases. A common clinical picture was not obvious in upd(6)mat, but intrauterine growth restriction (IUGR) and preterm delivery were frequent.

**Conclusion:**

A common upd(6)mat phenotype is not obvious, but placental dysfunction due to trisomy 6 mosaicism probably contributes to IUGR and preterm delivery. In fact, other clinical features observed in upd(6)mat patients might be caused by homozygosity of recessive mutations or by an undetected trisomy 6 cell line. Upd(6)mat itself is not associated with clinical features, and can rather be regarded as a biomarker. In case upd(6)mat is detected, the cause for the phenotype is identified indirectly, but the UPD is not the basic cause.

## Introduction

Uniparental Disomy (UPD) is the inheritance of the two homologous chromosomes of a pair from the same parent. It has meanwhile been reported for nearly all human chromosomes (reviewed in http://upd-tl.com/upd.html), and depending on the gene content of the affected chromosome there are three ways by which UPDs contribute to an aberrant phenotype:

- In case the same identical chromosome is inherited twice from the same parent (uniparental isodisomy, UPiD), homozygosity for an autosomal recessive mutation can occur. This cause of homozygosity for a recessive mutation has meanwhile been reported for numerous monogenetic disorders (reviewed in: Yamazawa et al. 2010) and the clinical picture is more or less specific for the disease.
- UPDs are often the result of a rescue mechanism in a trisomic zygote (“trisomic rescue”), and depending on the time the trisomic rescue occurs in the embryo it can be associated with trisomy mosaicism. In these situations, it is difficult to determine whether the aberrant phenotype is caused by the UPD itself or by the trisomic cell line. On molecular level, this mode of UPD formation is indicated by the presence of the two different homologues of a chromosomal pair from the same parent (uniparental heterodisomy, UPhD).
- If imprinted genes are affected (i.e., genes with monoallelical expression in a parent‐of‐origin‐specific manner) an imprinting disorder can occur.

The group of imprinting disorders currently comprises 12 entities (reviewed in: Soellner et al. 2017), and in the majority of them UPDs belong to the spectrum of molecular alterations. These include maternal UPDs of chromosomes 7, 11, 14, 15, and 20, and paternal UPDs of chromosomes 6, 11, 14, 15, and 20. Upd(6)pat is associated with (intrauterine) growth restriction and transient neonatal diabetes mellitus (TNDM) (Temple [Link]). In TNDM, approximately 40% of patients carry a upd(6)pat, but the molecular spectrum also comprises duplications of the paternal 6q24 allele or hypomethylation of the maternally methylated *PLAGL1* (*ZAC*) gene (OMIM 603044). The imprinted *PLAGL1* gene is over‐expressed in TNDM and encodes a DNA‐binding zinc‐finger protein that influences the expression of other genes (reviewed in: Gardner et al. 2000). A second chromosome 6 encoded gene associated with disturbed growth is *CUL7* (OMIM 609577), mutations in which lead to the 3M syndrome. *CUL7* has been reported to be paternally expressed in placenta (Hamada et al. 2016). Mice homozygous *Cul7*‐deficiency/deletions show intrauterine growth restriction (IUGR), and placentas are small. On the other hand heterozygous littermates exhibit a normal phenotype (Varrault et al. 2006).

In contrast to upd(6)pat, the clinical relevance of maternal UPD of chromosome 6 (upd(6)mat) is unclear, and only a limited number of cases have been reported (Table 1). The majority of them showed intrauterine growth restriction and preterm delivery, but other clinical features are not common. Here, we report on two new cases with upd(6)mat. Based on data from these patients and cases from the literature, we delineate the clinical significance of upd(6)mat.

**Table 1:** Overview on the upd(6)mat patients reported in the literature

Ref.	Reported Hetero‐/Isodisomy	Sex	Conventional karyotype	Method for UPD detection	Monogenic mutation	Placenta	Birth at	Cesarean section; reason (if known)	IUGR	PNGR	Age at last examination	Hernia	Failure to thrive	Further findings/comments
van den Berg‐Loonen et al. (1996)	Isodisomy	M	NR	STRs	–	NR	40 gw	NR	Yes		41 y	NR	No	Sarcoidosis, hypercalcemia
Spiro et al. (1999)	Isodisomy	F	NR	STRs	*CYP21*: p.I172N	Normal	37.5 gw	No	Yes	Catch‐up	2.65 y	NR	NR	Congenital adrenal hypoplasia pubarche, clitoral enlargement
Cockwell et al. (2006)	Heterodisomy	M	FISH: AF: 19% T. 16, CB: normal	STRs	–	Normal	(23 gw)	IUFD	No	NR		Yes	NR	Atrioventricular septal defect
Parker et al. (2006)	Hetero/Isodisomy	M	48,XXY,+mar[39]/ 47,XXY[20]	STRs	*CYP21*: deletion	NR	36 gw	Yes	Yes	Slight	8 m	NR	NR	Progressive respiratory distress due to persistent pulmonary hypertension,mild developmental delay
Hong et al. ([Link])	NR	NR	Prenatally detected trisomy 6	NR	–	NR	NR	NR	Yes	NR	NR	NR	NR	Ambiguous genitalia, persistent Mullerian structures
Gümüş et al. (2010)	Hetero/Isodisomy	M	NR	SNP array	*MOCS1*: p.R73W	NR	At term	Yes; no medical reason	No	NR	10 m	NR	Yes	Dandy‐Walker, seizures, microcephaly, developmental delay
Salahshourifar et al. (2010)	Heterodisomy	M	46,XY	STRs	–	NR	At term	No	No	No	2 y	NR	NR	Cleft lip (of other origin)
Sasaki et al. (2011)	Hetero/Isodisomy	M	NR	SNP array	*CUL7*: p.R992P	NR	36 gw	Yes	Yes	Yes	2.9/12 y	NR	Yes	3M syndrome
Poke et al. (2013)	Hetero/Isodisomy	F	46,XX	SNP array	–	Small	35 gw	Yes; IUGR	Yes	Catch‐up	35 m	NR	Yes	Global development delay, severe gastro‐esophageal reflux disease
Begemann et al. (2012b)	Heterodisomy	F	46,XX	SNP array	–	NR	34 gw	Yes; oligohydramnio, IUGR, poor cardiotocogram	Yes	Yes	7.5 m	Yes	Yes	Silver‐Russell syndrome, but caused by familial 11p15 duplication?
Roosing et al. (2013)	Isodisomy	NR	NR	SNP array	*TULP1:* p.R420S	NR	NR	NR	Yes	No	52 y	NR	NR	Cone dysfunction
Takimoto et al. (2015)	Isodisomy	F	NR	SNP array	–	NR	29 gw	NR	Yes	NR	6 m	NR	NR	WASP (X chr.): upd6 might be involved in the pathogenesis of XCI in females
Lazier et al. (2016)	Hetero/Isodisomy	M	46,XY	SNP array	–	NR	28 gw	Yes; IUGR	Yes	Yes	4 m	Yes	Yes	Abnormal genitalia, respiratory distress syndrome, persistent Mullerian structures
Leung et al. (2016)	Hetero/Isodisomy	F	Placenta: 47,XX,+6[12]/46,XX[19] AF: 46,XX	NR	–	NR	34 gw	Yes; oligohydramnio, IUGR	Yes	Too young		NR	NR	Increased amount of chromosome 6 material in maternal plasma fetal DNA
Leung et al. (2016)	Heterodisomy	F	Placenta: 47,XX,+6[14]/46,XX[16] CB: 46,XX	NR	–	NR	32 gw	Yes; reduced fetal movement, suboptimal cardiotocogram	Yes	Too young		NR	NR	
Case 1	Hetero/Isodisomy	F	46,XX (FISH: 98/100 normal, 2x monosomy 6)	SNP array	–	Normal	27 + 6	Yes; poor cardiotocogram	Yes	Yes	2.5 y	No	Yes	Facial dysmorphisms, clinodactyly of 5th digits, restlessness
Case 2	Hetero/Isodisomy	M	46,XY	SNP array	*CYP21:* deletion	NR	30 gw	Yes; oligohydramio, IUGR	Yes	Yes	3.8/12 y	No	NR	AGS, facial dysmorphisms, clinodactyly of 5th digits, flat valgus feet

NR, not reported; IUFD, intrauterine fetal death; STRs, short tandem repeats; SNP, single‐nucleotide polymorphism; gw, gestational week; y, year; m, month; AF, amniotic fluid; CB, chord blood; AGS, adrenogenital syndrome.

## Materials and Methods

### Molecular testing

Screening for molecular alterations of the *PLAGL1* differentially methylated regions (DMRs, *PLAGL1*:alt‐TSS‐DMR and *IGF2R*:Int2‐DMR) in 6q24/(6q25) is implemented in our routine diagnostic testing for growth retarded patients referred with clinical features of Silver‐Russell syndrome (SRS) and more than 1000 patients have meanwhile been tested. The results in 571 have been reported previously (Eggermann et al. 2014). The study was approved by the Ethical committee of the University Hospital Aachen, Germany.

**Figure 1:**
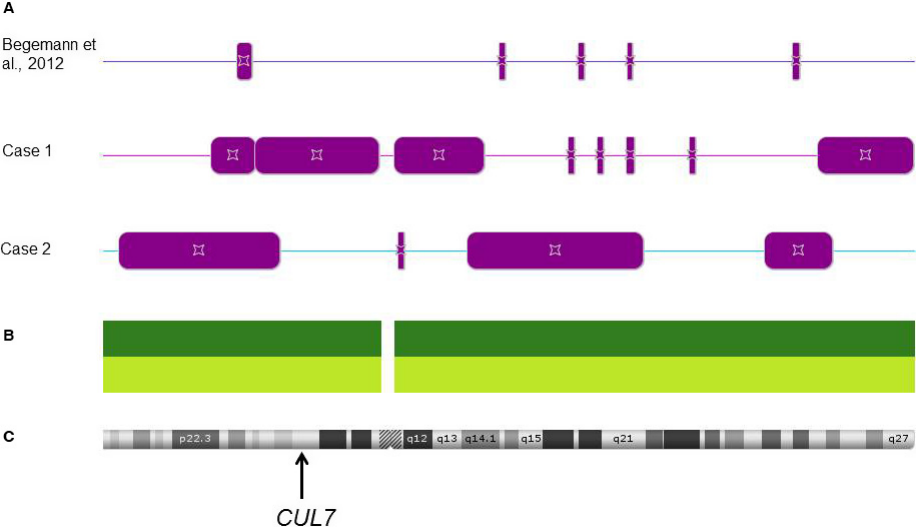
SNP array analyses (CytoScan, Affymetrix) of three upd(6)mat patients reveal regions of homozygosity which correspond to UPiD (CytoScan results are analyzed with the ChasSoftware, Affymetrix, Wycombe/UK). (A) Distribution of stretches with loss of heterozygosity (bars) corresponding to isodisomic uniparent disomy regions. (B) Distribution of SNP and CNV probes on the array. (C) Ideogram of chromosome 6 and rough localization of the *CUL7* gene.

Molecular testing comprised methylation‐specific (MS) single‐nucleotide primer extension (MS‐SNuPE) (Begemann et al. 2012a) and/or MS multiplex ligation probe‐dependent amplification (MS‐MLPA; ME030, ME032, ME034 from MRC Holland, Amsterdam/NL). In the two upd(6)mat cases (Cases 1 and 2) reported in this paper, the coding sequence of the *CUL7* gene (NM_001168370) was Sanger sequenced according to standard protocols. To discriminate between isodisomic and heterodisomic UPD regions and to exclude copy number variations in the two new patients and that of Begemann et al. (2012b), SNP array analysis was performed (Cytoscan, Affymetrix, Wycombe/UK) (Fig. 1).

Methylation analysis of the *CUL7* DMR described by Hamada et al. (2016) was conducted in DNA from blood of normal controls, three first and three third‐term placenta samples (Grothaus et al. 2016) (Beygo et al., submitted; maternal contamination was excluded before) by next‐generation bisulfite sequencing on the Roche/454 GS junior system (Branford, CT, USA) as described previously (Beygo et al. 2013) using tagged primers F1 5′‐CTTGCTTCCTGGCACGAG‐GGGTAGGGTGTATAGATTAGGTAGG‐3′ with R1 5′‐CAGGAAACAGCTATGAC‐CCCTTACTCTATAAAAAACAAACCTC‐3′ and F2 5′‐CTTGCTTCCTGGCACGAG‐GAGGTTTGTTTTTTATAGAGTAAGGGA‐3′ with R2 5′‐CAGGAAACAGCTATGAC‐TCCAAAATCTTTTCCAATTCTAACTT‐3′.

### Literature query

A literature search was conducted using Pubmed and the search terms “uniparental disomy” and “chromosome 6”. Thereby we identified 15 cases for which we determined the following parameters (whenever possible), including that of Begemann et al. (2012b): gender, reported hetero/isodisomy, karyotyping results, method of UPD detection, pathogenic genomic variants, result of the macroscopic investigation of the placenta, intrauterine and postnatal growth, age at diagnosis and/or last examination, hernia, and failure to thrive.

In those cases where SNP array data were available (new cases, Begemann et al. 2012b; Gümüş et al. 2010; Roosing et al. 2013; Sasaki et al. 2011), we searched for common isodisomic regions.

### Case 1

The patient is the first child of healthy unrelated parents (maternal age at birth: 36 years, paternal age: 44 years). Intrauterine growth restriction was observed at gw 25, and malfunction of the placenta was reported. Pathological cardiotocography (CTG) records showed fetal bradycardia and led to caesarean section at 27 + 6 gestational week (gw). Histopathological investigation of the placenta showed a disturbed differentiation without signs of inflammation. Weight at birth was 650 g (−1.49 SD), length 35 cm (−0.42 SD), head circumference 23 cm (−1.54 SD). Apgar scores were 7/9/9.

After birth, growth restriction persisted: At the age of 2 7/12 years height was 82 cm (−2.66 SD), and head circumference 46 cm (−3.22 SD).

Facial dysmorphisms included large, simple ears, a long, slightly triangular face, frontal bossing, large eyes, and a prominent chin. Clinodactyly of the fifth digits was present. Body asymmetry, further dysmorphisms or malformations were not observed.

No complications were reported in the newborn period, but restlessness and short sleep periods were noticed. Psychomotor development was within the normal range, speech development was mildly delayed.

By MS‐MLPA and MS SNuPE, hybridization corresponding to a hypermethylation of the *PLAGL1* and *IGF2R* DMRs could be identified. Subsequent typing of chromosome 6 microsatellite markers confirmed upd(6)mat, a discrimination between isodisomic and heterodisomic regions became possible by SNP array analysis (CytoScan, Affymetrix, High Wycombe/UK). By the same approach pathogenic deletions or duplications of >50 kb were excluded. As the *CUL7* gene was localized in one of the isodisomic segments, its coding region was sequenced but sequencing data did not exhibit any pathogenic mutation. Conventional karyotyping in peripheral lymphocytes at the age of 2 4/12 years revealed a normal 46,XX karyotype. FISH analysis with a chromosome 6 probe (CEP 6, Locus D6Z1; Abbott, Illinois/USA) in 100 buccal mucosa cells did not provide any evidence for a trisomy 6 mosaicism.

### Case 2

The patient is the fifth child of healthy unrelated parents (maternal age at birth: 41 years, paternal age: 45 years). His other siblings were healthy. The pregnancy was unremarkable until gw 30 when oligohydramnios and an intrauterine growth restriction were observed. Because of fetal distress, the patient was delivered by caesarean section at gw 30 + 6. Weight at birth was 1100 g (−0.68 SD), length 38 cm (−1.07 SD), and head circumference 28 cm (−0.25 SD). Apgar scores were 7/8/9. Artificial ventilation was required after birth, and the boy stayed in hospital for 2 months. He had congenital adrenal hyperplasia (AGS) due to 21‐hydroxylase deficiency caused by a homozygous deletion affecting exons 1–8 of the *CYP21A2* gene on 6p21.3. As this finding did not explain the persisting growth restriction, the boy was referred for genetic counseling at the age of 3 8/12 years. At that time his body measurements were all below the 3rd percentile (height 91 cm (−3.34 SD), weight 12 kg (BMI 14.5), head circumference 48.5 cm (−2.18 SD)). Further dysmorphic features included short palpebral fissures, small nasal wings and lips, relatively large ears, clinodactyly V, and flat valgus feet.

Conventional cytogenetic analysis in peripheral lymphocytes revealed a normal male karyotype (46,XY). By MS‐MLPA, normal methylation patterns were observed for differentially methylated regions (DMRs) on chromosomes 11p15 (*IGF2/H19*), 7p12 (*GRB10*), and 7q32 (*MEST*) but a hypermethylation could be detected for the *PLAGL1* locus on chromosome 6. Gene dosage analysis for *PLAGL1* was normal, therefore a sporadic imprinting defect or a upd(6)mat was suggested. However, the homozygous deletion of the *CYP21A2* gene indicated a upd(6)mat. Unfortunately, DNA from the father was not available, but by combining all molecular data upd(6)mat was concluded. Accordingly, SNP array analysis (CytoScan, Affymetrix, High Wycombe/UK) showed large regions with isodisomy for chromosome 6. Pathogenic CNVs >50 kb could not be detected. Sanger sequencing of the coding region of the *CUL7* gene was negative, the detection of a heterozygous SNP in the gene revealed that the boy is not isodisomic for the *CUL7* locus.

## Results

In the course of routine molecular analysis in patients with congenital growth restriction at the Institute of Human Genetics Aachen, more than 1000 samples were analyzed. In addition to the differentially methylated regions on chromosomes 7, 11, and 14 which are affected in the imprinting disorders SRS and Temple syndrome, imprinted loci (*PLAGL1, IGF2R*) on chromosome 6 were investigated in all these cases as well. By this approach, we detected one new patient with a upd(6)mat (Case 1). A second patient with upd(6)mat (Case 2) was identified at the Institute of Human Genetics in Essen.

SNP array analyses in these two new cases and a third upd(6)mat patient published previously (Begemann et al. 2012b) indicated both heterodisomic and isodisomic regions on chromosome 6 (Fig. 1). The comparison of the isodisomic segments in these cases with those from the literature (Gümüş et al. 2010; Sasaki et al. 2011; Roosing et al. 2013) did not reveal a common isodisomic region, even when the patient without IUGR (Gümüş et al. 2010) was removed from the analysis.

The compilation of molecular data from all 17 published cases with upd(6)mat (Table 1) showed that the majority of cases were heterodisomic or carried both heterodisomic and isodisomic segments. Conventional karyotyping revealed a normal karyotype in the five patients with postnatal lymphocyte analysis, whereas in four prenatally identified cases, a trisomy 6 mosaicism was detected. In four patients, homozygosity for recessive mutations in genes on chromosome 6 was identified, resulting in disease‐specific phenotypes. A common clinical picture was not obvious in the upd(6)mat patients, but the majority showed a IUGR and/or a preterm delivery.

**Figure 2:**
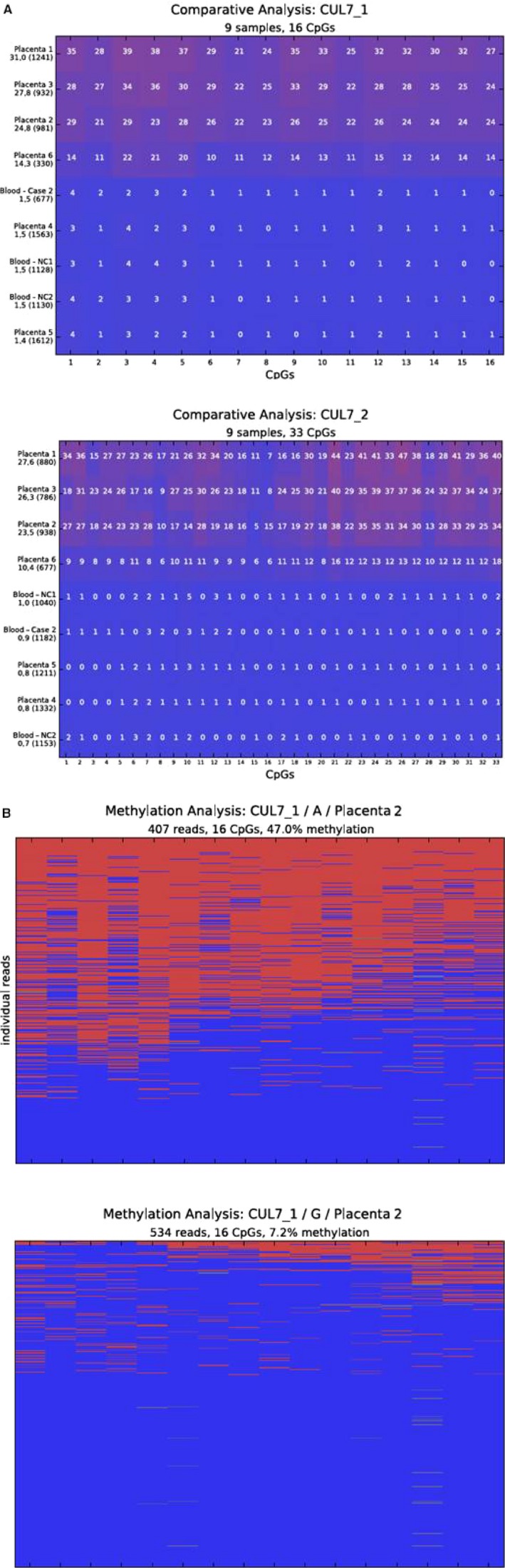
Results of the methylation analyses by deep bisulfite sequencing. (A) Comparative results for both amplicons covering the *CUL7*‐DMR. Each sample is represented in a single line. The average methylation over all analysed CpGs is given below the sample name on the left hand side together with the number of analysed reads. Every square represents an analysed CpG. The number inside gives the average methylation of the CpG over all analysed reads of the sample. Red is methylated, blue is unmethylated. (NC ‐ normal control blood sample; Case 2 – Case 2 blood sample; Placenta 1‐3 – first‐term placenta samples; Placenta 4‐6 – third‐term placenta samples). (B) Methylation result for the informative first‐term placenta 2 sample. The figure shows the result of the methylation analysis for the sample after allele separation using the informative SNP rs55890439. Methylation for allele A are displayed in the plot on the left and for allele G on the right. Each line represents a single read, each column a CpG. The number of analysed reads, the average methylation over all analysed reads, and CpGs as well as the allele is given above the plot. Red is methylated, blue is unmethylated.

We analyzed a large part of the *CUL7* DMR described by Hamada et al. (2016) using two amplicons for deep bisulfite sequencing. In DNA from blood of normal controls, we found that all investigated 49 CpGs were unmethylated (Fig. 2A). In three first‐term placenta samples, methylation levels of 20–30% were determined (Fig. 2A). In one of these samples, we could discriminate the alleles by utilizing an informative SNP (Sample 2, Fig. 2B). One allele revealed a methylation of 7.2%, while the other allele showed a preferential methylation with 47.0%. For the three third‐term placenta samples, we detected about 10–14% methylation in one sample, while the other two samples are unmethylated (Fig. 2A). We also investigated DNA from blood of patient 2 but like in the normal controls, methylation could not be detected.

## Discussion

Maternal and paternal UPDs have been reported for nearly all human chromosomes, and for the majority of them the clinical significance is known. Many UPDs are not associated with a specific phenotype but are only detected in case of a homozygosity for a recessive mutation. However, there are some maternal and/or paternal UPDs which disturb the balanced expression of imprinted genes and thereby cause imprinting disorders.

Whereas is it out of question that upd(6)pat is associated with TNDM, the clinical findings are heterogeneous. So far, only 15 cases with upd(6)mat have been recorded (Table 1), and we now add clinical and molecular data from additional two cases. Overall, a common phenotype was not obvious among the upd(6)mat cases, with the exception of IUGR and preterm delivery. However, these features are unspecific and observable in several congenital disorders, including chromosomal aberrations.

Among all 17 upd(6)mat patients, five were homozygous for recessive mutations, and exhibited the respective phenotype (e.g., 3M syndrome, AGS; Table 1). In 2013, Poke and colleagues (Poke et al. 2013) suggested that homozygosity of an autosomal recessive mutation in 6q16.1qter might cause some clinical features of the condition, or at least for IUGR. However, the comparison of the available SNP data in upd(6)mat patients (*n* = 6) reveals that there is no overlap of isodisomic regions. Thus, a common autosomal recessive gene defect is obviously not the cause for clinical features in upd(6)mat.

The unbalanced expression of imprinted genes on chromosome 6 has been regarded as another explanation for upd(6)mat phenotypes. A striking candidate gene on chromosome 6 is *PLAGL1* (*ZAC*): Knock‐out experiments in mice reveal that *Zac*+/− (pat) puppets are growth retarded, whereas Zac+/−(mat) mice are of normal growth (Varrault et al. 2006). As upd(6)mat functionally corresponds to a deletion of the active paternal allele, the findings in knock‐out mice might explain the IUGR in patients with upd(6)mat. However, the precise function of *PLAGL1* is currently unknown, and the finding of IUGR also in upd(6)pat rather indicates that altered imprinting marks are not the cause of IUGR in case of upd(6). Another gene which is reported to be only paternally expressed in the placenta is *CUL7* (Hamada et al. 2016). By methylation analyses in placenta samples, we could not support the suggested imprinted status of *CUL7*. Our data show that the *CUL7* DMR is only partially methylated in human first‐term placentas and mostly unmethylated in third‐term placentas.

Finally, the pathoetiological association of IUGR with upd(6)mat became with the reports of of two upd(6)mat patients with normal growth parameters [(Gümüş et al. 2010): 3705 g (75th P), length 50 cm (50th P), head circumference 35 cm (50th P); Salahshourifar et al. (2010): 3.700 g (75th P), 50 cm (50th P), OFC 35 cm (50th P)] the head circumference (OFC) was in the normal range (50th P).

Another frequent finding in upd(6)mat pregnancies is (induced) preterm delivery (10/12 cases). Interestingly, it is also reported in other imprinting disorders, but systematic studies to determine its frequency and to uncover the causes for this feature.

Although homozygous autosomal recessive mutations or disturbed imprint marks on chromosome 6 are not causative for a specific upd(6)mat phenotype, increasing evidence indicates that the clinical features in upd(6)mat patients are caused by an (undetected) trisomy 6 mosaicism. This mosaicism can be present either in the patient himself, or it can be confined to the placenta. The latter would explain why only IUGR is present in some upd(6)mat individuals, whereas postnatal growth is normal. Trisomy 6 itself is not viable, but a few cases of trisomy 6 mosaicism have been identified prenatally (for review: Gardner et al. 2012). In these cases, the fetal features ranged from minor to severe, but normal outcomes have also been reported (Hsu et al. 1997). Therefore, a correlation between the level of mosaicism and the phenotype has been suggested. There is one follow‐up report on a liveborn with trisomy 6 mosaicism (Miller et al. 2001). Prenatal ultrasound exhibited several ultrasonographic anomalies, and in chorionic villous sampling trisomy 6 could be identified (60% in short‐term, 22% in long‐term culture). After birth, trisomy 6 was confirmed in skin fibroblasts (3–20%), whereas the karyotype in blood was normal. Clinical follow‐up at an age of 2 3/4 years revealed a growth restriction (<P3), neurodevelopment was normal. These observations are compatible with the data from conventional karyotyping in the upd(6)mat cohort. In 11 patients, cytogenetic/FISH analyses were performed and gave a normal karyotype in peripheral lymphocytes in six of them, in one patient a 47,XXY constitution was present. In four cases, prenatal testing was carried out, and trisomy 6 mosaicism was detected in all of them (Table 1). In fact, the presence of trisomy 6 mosaicism in the two new cases reported here could not be confirmed as only lymphocytes could be analyzed. Nevertheless, the increased maternal ages correspond to the UPhD in both cases and its underlying formation mechanism.

In conclusion, these data show that (placental) trisomy 6 mosaicism contributes to IUGR, whereas the other heterogeneous clinical features in upd(6)mat patients are either caused by undetected trisomy 6 cell lines or by homozygosity for recessive mutations (Spiro et al. 1999; Parker et al. 2006; Gümüş et al. 2010; Sasaki et al. 2011; Roosing et al. 2013). Upd(6)mat itself does not cause clinical features, but can be regarded as a biomarker, comparable to maternal UPD of chromosome 16 (Scheuvens et al. 2016). However, in case upd(6)mat is detected in patients with unspecific clinical features, it is assumable that the cause for the phenotype is identified.

Finally, the identification of a upd(6)mat patient by a routine multilocus screen for imprinted loci confirms the power of this approach. Even rare conditions, like upd(6)mat or upd(20)mat, are detectable by this comprehensive approach in patients suspected to suffer from an imprinting disorder (i.e., growth‐retarded patients with SRS features).

## Conflict of Interest

The authors disclose any commercial association that might pose or create conflict of interest with the information in this manuscript.
